# The Cost-Effectiveness of Lowering Permissible Noise Levels Around U.S. Airports

**DOI:** 10.3390/ijerph14121497

**Published:** 2017-12-02

**Authors:** Boshen Jiao, Zafar Zafari, Brian Will, Kai Ruggeri, Shukai Li, Peter Muennig

**Affiliations:** 1 Global Research Analytics for Population Health, Columbia University Mailman School of Public Health, New York, NY 10032, USA; zz2492@cumc.columbia.edu (Z.Z.); dr2946@cumc.columbia.edu (K.R.); lishukai@pku.edu.cn (S.L.); pm124@cumc.columbia.edu (P.M.); 2 Queens Quiet Skies, Bayside, NY 11360, USA; brian.f.will@gmail.com

**Keywords:** cost-effectiveness, aircraft noise, regulatory change, sound insulation

## Abstract

Aircraft noise increases the risk of cardiovascular diseases and mental illness. The allowable limit for sound in the vicinity of an airport is 65 decibels (dB) averaged over a 24-h ‘day and night’ period (DNL) in the United States. We evaluate the trade-off between the cost and the health benefits of changing the regulatory DNL level from 65 dB to 55 dB using a Markov model. The study used LaGuardia Airport (LGA) as a case study. In compliance with 55 dB allowable limit of aircraft noise, sound insulation would be required for residential homes within the 55 dB to 65 dB DNL. A Markov model was built to assess the cost-effectiveness of installing sound insulation. One-way sensitivity analyses and Monte Carlo simulation were conducted to test uncertainty of the model. The incremental cost-effectiveness ratio of installing sound insulation for residents exposed to airplane noise from LGA was $11,163/QALY gained (95% credible interval: cost-saving and life-saving to $93,054/QALY gained). Changing the regulatory standard for noise exposure around airports from 65 dB to 55 dB comes at a very good value.

## 1. Introduction

In the United States, the allowable limit for sound in the vicinity of an airport is 65 decibels (dB) averaged over a 24-hour ‘day and night’ period (DNL) [1]. This level is roughly 10 times the sound intensity (measured in power) of a 55 dB day–evening–night level (L_den_), the threshold in the European Union. The actual difference in noise in the U.S. can be higher because dBs are measured on a logarithmic scale, and DNL levels are weighted to increase the impact of nighttime noise. This high level of sound exposure can affect sleep, well-being, school performance, and economic productivity as well as mental and physical health [2,3,4,5,6]. It has also been shown in observational and quasi-experimental analyses to produce an increase in risk of cardiovascular disease (CVD) and mental illness [7,8,9,10,11].

Airports tend to have sound contour patterns that correspond to aircraft approach, departure, size, and geographic characteristics [2,3]. Under Part 150 of the Federal Aviation Regulations, 65 dB DNL is considered “significant”, and houses that fall within noise contours that exceed allowable limits must receive remediation by the government.

A very large study of aircraft noise corridors in the United Kingdom showed that 55 dB is the threshold value at which mental health problems emerge [12]. Similar findings in Europe led to the EU’s Environmental Noise Directive [8,13], which limits aircraft noise corridors to 55 dB L_den_ [14].

Aircraft noise abatement measures are in effect around the world [15], and can reduce the noise impact on population [16]. The International Civil Aviation Organization (ICAO) established a “balanced approach” to aircraft noise management, including four elements: “reduction of noise at its source; land use planning and management; noise abatement operational procedures; and operating restrictions on aircraft” [17]. The characteristics of a given airport, such as the number of runways, the number of aircraft movements, the type of aircraft, the population of the city it serves, the per capita gross domestic product of the country an airport is located in, can inform the ideal abatement strategy [18,19]. Options for addressing noise at its source (e.g., altering the runway characteristics, changing flight patterns, reducing aircraft size, requiring aircraft to glide, and altering climbs on departure) are both more effective and generally less expensive than land use planning and management (e.g., land acquisition and sound insulation); however, the latter are likely inevitable in most localities in the United States if more stringent standards are to be adopted [17,20]. This is because many of the traditional means of reducing noise at its source have already been implemented, and more recent innovations that consider both airport and aircraft characteristics are challenging to implement due to conflicting air traffic at adjacent urban airports [21].

Still, land use changes carry their own challenges. While aircraft noise reduces the price of property by approximately 20% on average [22], buying properties via eminent domain is prohibitively expensive in most urban areas in the United States. Because further source mitigation is unlikely to reduce 65 dB DNL contours to 55 dB DNL, most localities would likely resort to home insulation against sound as a strategy for reducing noise exposure among individuals.

The much weaker regulatory standard in the United States means that authorities or municipalities can maintain or expand airports in the United States more cheaply than can European governments. However, this weaker standard may also harm the health of those who live near airports. This paper examines the trade-off between the direct cost of changing the regulatory DNL level from 65 dB to 55 dB versus the medical costs, loss of health, and loss of lives associated with failing to do so. We examine sound insulation as a noise abatement strategy because it is more expensive and less effective than most noise source abatement strategies—if sound insulation is cost-effective, then it is not necessary to evaluate noise source abatement strategies because they are intuitively cost-effective.

## 2. Materials and Methods

### 2.1. Overview

**Figure 1:**
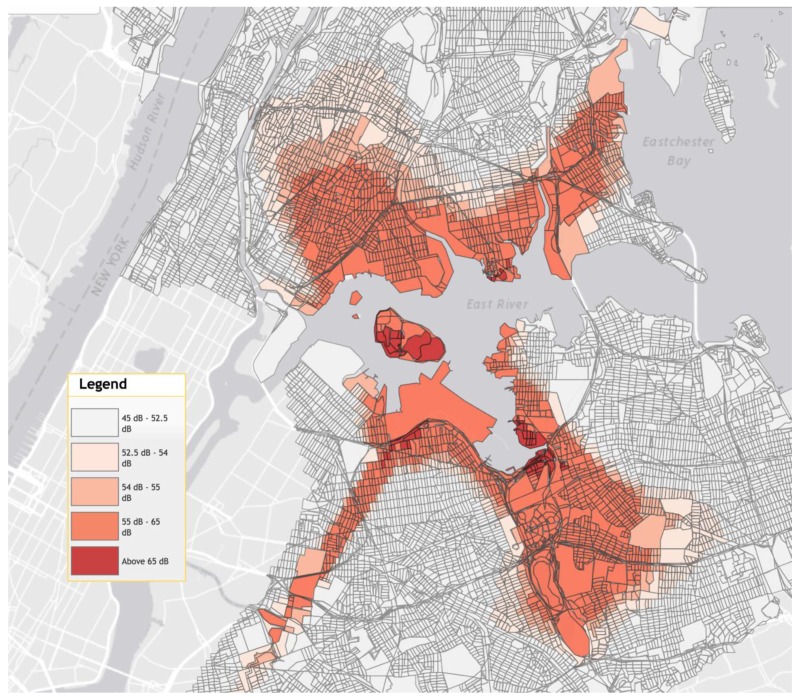
Noise levels around La Guardia Airport. Source: MSP FairSkies Coalition and University of Minnesota Center for Urban and Regional Affairs, 2010 [23].

The cost-effectiveness of installing sound insulation to residential homes is evaluated using a residential housing area around LaGuardia Airport (LGA) in New York City (NYC) as a case study. We chose this area as a case study because there are good data on both the noise exposure and the number of households impacted by them. The map of aircraft noise levels developed by the Minneapolis-Saint Paul (MSP) FairSkies Coalition and the University of Minnesota Center for Urban and Regional Affairs are presented in Figure 1 [23]. Housing exposed to 55 to 65 dB DNL was mostly built in the immediate post World War II era. This precedes the commercial use of modern jets at the airport and thus housing within the contours contains very little heat or sound insulation [24]. If it is cost-effective to insulate homes near LGA, then it is likely a cost-effective strategy everywhere. If it is not cost-effective to insulate homes near LGA, then local-area analyses would be needed on a case-by-case basis.

To reflect the lifetime health and economic impact of sound insulation, a Markov model was built. The model follows a hypothetical cohort with an average age of 36 years (the median age in NYC) to estimate costs and health outcomes associated with two scenarios [25]:

(1) People currently being exposed to >55 to ≤65 dB DNL;
(2) The same cohort with noise insulation adequate to reduce noise exposure to below 55 dB DNL.

A societal perspective was assumed, and included the cost of sound insulation; the medical costs associated with CVD and anxiety; and indirect costs, such as lost productivity. The quality-adjusted life year (QALY) was used as a health outcome measure. One QALY is equal to a year of life lived in perfect health. Cost-effectiveness outcomes were measured in terms of incremental cost-effectiveness ratio (ICER), or the change in costs divided by the change in QALYs when sound insulation is installed in homes. Willingness-to-pay (WTP), defined as the maximum price the society is willingness to pay for one QALY gained, offers information on the monetary value of a QALY gained [26]. We employed a WTP of $50,000 per QALY gained, which is considered to be a very conservative price for a QALY in the United States [27]. From a financial standpoint, an intervention that costs less than $50,000/QALY gained would be considered a very good value. A 3% discount rate was used for future costs and health outcomes in concordance with recommendations of the Panel on Cost-effectiveness in Health and Medicine [28]. The model was built in TreeAge Pro 2016.

For simplicity, we assumed that the commonly exposure measures in different jurisdictions (e.g., DNL, Lden, Ldn, and CNEL) are similar. In practice, the DNL standard in the United States is less strict because it does not add a penalty for evening flights.

### 2.2. Model Parameters

#### 2.2.1. Probability

The probability parameters can be found in Table 1. The simulated participants were exposed to a background risk of CVD, anxiety, or death. The age-specific probability of developing a CVD event for adults above 35-years-old was derived from a report provided by the Heart and Stroke Association Statistics [29]. The probability of anxiety disorder was obtained from the U.S. National Comorbidity Survey [30]. In addition, background mortality rates were obtained from U.S. Life Tables [31].

#### 2.2.2. Relative Risk

Exposure to 55 dB DNL or above is associated with a 12% higher risk of CVD (14% for daytime noise and 9% for night time noise) and a 69% higher risk of an anxiety disorder in the average person (See Table 1) [8,9]. For those with a prior versus no history of CVD, the relative risks of developing a new CVD-related event and anxiety are 1.96, and 1.66, respectively [32,33].

#### 2.2.3. Cost

The average nation-wide cost of sound insulation was estimated from a study by the Wolfe et al. (while NYC is a case study, average costs were used so that the marginal costs and effects could be generalized to other cities within the United States) [20]. The life of installed sound insulation is estimated to be 25 years, as it is susceptible to degradation and moisture intrusion, and this is also roughly the usable life of modern sound insulating windows [34].

Medical and work loss costs associated with anxiety were obtained from the U.S. comorbidity survey [35]. The medical costs of CVD were derived from a study based on the Kaiser Permanente Northwest CVD registry [32]. The productivity loss due to CVD was estimated to be about 55% of the medical cost [36]. All monetary costs were adjusted to 2016 U.S. dollars using the Consumer Price Index of U.S. and New York (Table 1).

#### 2.2.4. Health Utility

Health utilities, which are preference weights for different health status, are needed for calculation of QALYs [37]. Health utilities are measured on a scale ranging from 0 (death) to 1 (full health) [37]. The health utility values associated with new CVD and prior CVD history from published studies were obtained using EuroQol-5 Dimension (EQ-5D) [38,39]. The EQ-5D-3L has five dimensions: mobility, self-care, usual activities, pain/discomfort, and anxiety/depression [40]. All of these dimensions are scored based on a three-point scaling system, ‘1’ representing no problems, ‘2’ representing moderate problems, and ‘3’ representing severe problems. The utility value of anxiety was calculated based on a one-point increase (from 1 to 2) in a three-point scale of the anxiety/depression dimension of the EQ-5D, while making no change in the other dimensions (Table 1) [41].

### 2.3. Decision Analysis Models

The main assumptions used in the Markov model are:

(1) We used the average nation-wide cost of sound insulation per person for our model. It was assumed that the household size in neighborhoods near LGA was same as nation-wide average household size.
(2) The RRs of CVD and anxiety associated with aircraft noises were based on studies conducted in the United Kingdom and Italy. We assumed that they were generalizable to the United States.
(3) There was no data on RRs of CVD and anxiety associated with aircraft noises for different age groups. They were assumed to be constant over the life course.
(4) For those who developed both CVD and anxiety, we assumed that health utility decrement due to anxiety was already captured in health utility decrement due to CVD.

**Figure 2:**
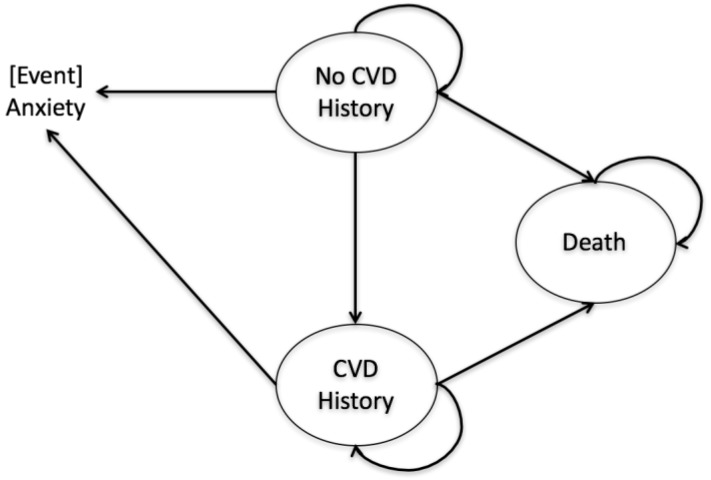
Markov model diagram.

The Markov model had three health states: no CVD history, CVD history, and death. The model diagram is presented in Figure 2. CVD was defined as codes I00 to I99 in the 10th Revision of the International Classification of Diseases, and was consistent across model inputs [29]. Any simulated participant death from CVD or other causes is transitioned to death as an absorbing health state. If a simulated participant currently in the “no CVD history” state developed a CVD-related event, he/she would transition to the state “CVD history” in the next cycle, and will stay there until he/she dies (either of CVD or other causes). In addition, in each cycle there is a chance of developing an anxiety disorder for each simulated participant in the model.

Health states include: no cardiovascular disease (CVD) history, CVD history, and death. Anxiety was included as an event instead of a health state.

A series of one-way analyses along with a probabilistic sensitivity analysis (10,000 Monte Carlo simulations) were conducted to assess the uncertainty within the model.

## 3. Results

The main results of the cost-effectiveness analysis are presented in Table 2. Over the course of one’s life, installing sound insulation is associated with approximately $6793 in increased costs to society and an increase in 0.61 QALYs gained. The resulting incremental cost effectiveness ratio (ICER) of the intervention relative to status quo was, therefore, $11,163/QALY.

Table 3 lists the effects of a series of one-way sensitivity analyses on ICERs. The most sensitive input parameter in the model was the relative risk of anxiety for noise exposure above 55 dB DNL. If it was assumed that there was no increased risk of anxiety due to aircraft noise, the ICER would increase to $195,717/QALY gained. Among costs parameters, the intervention cost was the most influential. Increasing the intervention cost by 25% increased the ICER to $24,448/QALY gained from its base case value (i.e., $6793/QALY gained). Additionally, when the probability of developing CVD was increased by 25%, the ICER dropped to $7064/QALY. Other model parameters did not show any significant effect.

**Figure 3:**
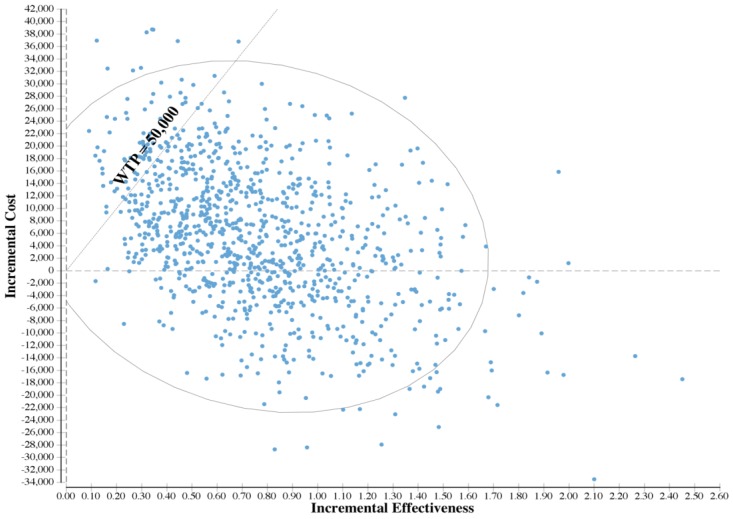
Incremental cost-effectiveness scatter-plot, changing the regulatory DNL level from 65 dB to 55 dB versus the status quo.

Results indicate a 95% credible interval of the ICER ranging from dominance to as high as $93,054/QALY gained. The incremental cost-effectiveness plane is shown in Figure 3. Based on this figure, installing sound insulation would be cost-effective in 91% of simulations at a WTP value of $50,000/QALY gained.

The dots represent incremental cost-effectiveness pairs of Monte Carlo simulations for changing the regulatory DNL level from 65 dB to 55 dB versus the status quo for 10,000 Monte Carlo simulations. The diagonal line represents a WTP of $50,000 per QALY gained. The dots to the right of the diagonal line, of which the proportion is 91%, represent the simulations with an incremental cost effectiveness ratio less than $50,000 per QALY gained.

## 4. Discussion

In the United States, the Environmental Protection Agency’s Office of Noise Abatement and Control was shuttered in the 1980s [42]. This office had successfully restricted noise at one airport in the name of public health [42], but failed to have a national impact on standards. As a result of generally weaker regulatory standards for noise, the burden of regulation has fallen on the Federal Aviation Administration (FAA), an agency focused on regulating lives in the air rather than on the ground.

However, while between 600 and 1300 people die in aviation accidents worldwide [43], most deaths probably occur on the ground from day-to-day airport operations. A risk ratio of 1.12 for CVD amounts to 45 excess deaths per 100,000 people exposed to DNL > 55 dB [29]. It is likely that many millions are exposed to airplane noise that exceeds these levels worldwide, mostly in developing nations and in the United States.

This study illustrates the risks associated with a failure to rationally allocate resources to maximize lives saved costs the lives of tens of thousands of Americans each year [44,45]. Changing the standard for DNL from 65 dB to 55 dB in the United States also comes at a cost that is one to two orders of magnitude lower than the cost of most FAA regulations geared toward saving lives in the air [45,46]. The air marshal program, for example, costs $180 million per life saved [47]. It is also more cost effective than the vast majority of medical and health interventions that experts agree are worthwhile investments. For instance, screening and treating high-risk populations for HIV comes in at over four times the cost of the lowering the DNL standard in the United States [48]. The Monte Carlo simulations indicate that 91% of the permutations of the model would cost under $50,000/QALY gained, which is similar in cost to HIV screening and treatment in high-risk populations.

The current study was subject to a number of limitations. The relative risk estimates of CVD and anxiety for aircraft noise exposure were only based upon observational studies. This is because existing experimental and quasi-experimental studies generally include heart rate and a psychological rating scale as outcome measures, both of which are not suitable with the current model [10,49]. However, these risk estimates are in line with the observational data.

A second potential weakness of the study is the use of a case study neighborhood. While necessary to generate estimates for the purposes of illustration, the use of a case study limits the generalizability of our findings. However, the East Elmhurst neighborhood consists of relatively densely packed private houses with poor insulation and relatively low DNL exposures within sound corridors [24]. Areas with high-rise buildings or neighborhoods near airports that support larger, long-distance aircraft could prove challenging to insulate to ≤55 dB DNL. The United States also regulates housing more stringently and has far higher health costs than less wealthy nations, further reducing the generalizability of our findings to other development contexts.

Third, the CVD noted in this study is likely to arise from anxiety associated with airplane nose. Still, we treated anxiety and CVD as independent events. We chose to do so because the nature of each condition is quite different, and would likely lead to separate, independent diagnoses in very different settings. Anxiety can influence work productivity and incur outpatient costs. CVD likely influences inpatient costs to a much greater extent. However, it may be that patients with co-morbid anxiety and CVD patients incur higher than average CVD costs (e.g., by consuming more hospital time).

Finally, it should be noted that sound insulation is an imperfect remedy. While it reduces noise in the home, outdoor activities, such as gardening, are made considerably less pleasant. Moreover, people are likely less willing to open the windows of their homes, also influencing the quality of their lives. These costs were not considered in the analysis.

## 5. Conclusions

Many strategies exist for abating aircraft noise, including reducing noise at the source (e.g., changes to flight patterns) and reducing noise exposure for those on the ground (e.g., insulation of homes). Of these strategies, insulating homes is likely to be both more expensive and less effective than source strategies. Nevertheless, we find that lowering the allowable limit of aircraft noise from 65 dB to 55 dB DNL would be cost-effective, even if it requires insulating homes near airports. This is because the cost of inaction is very high, taking a toll on the mental and physical health of those living near airports. Indeed, even when this strategy is adopted, changing the regulatory standard for noise exposure around airports comes at a very reasonable cost given the significant benefits for health.

It is important for agencies like the FAA to set more rigorous standards that take into account the significant health impacts for those on the ground.

## Figures and Tables

**Table 1:** Values used in the Markov model evaluating changing the regulatory day–night average sound level (DNL) from 65 decibels (dB) to 55 dB versus the status quo.

Parameter	Base	Standard Error/Range	Distribution *	Source
**Cost (2016 U.S.$)**				
Direct Cost				
Medical cost of cardiovascular disease (CVD)	23,229	5807	Gamma	Nichols et al., 2010
Medical cost of anxiety	2814	704	Gamma	Greenberg et al., 1999
Cost of sound insulation	18,959	4740	Gamma	Wolfe et al., 2016
Indirect Cost				
Productivity loss of CVD	12,837	3209	Gamma	Lloyd-Jones et al., 2010; Nichols et al., 2010
Productivity loss of Anxiety	313	78	Gamma	Greenberg et al., 1999
**Health Utility Value**				
Health utility decrement due to CVD	0.283	0.0130	Beta	Ara et al., 2010; Ara et al., 2009
Health utility decrement due to anxiety	0.156	0.0391	Beta	EQ5D
Health utility of CVD history	0.844	0.0096	Beta	Ara et al., 2010; Ara et al., 2009
**Probability**				
Probability of developing a CVD (by age)				Mozaffarian et al., 2016
35–44	0.15%	0.04%	Beta	
45–54	0.71%	0.18%	Beta	
55–64	1.49%	0.37%	Beta	
65–74	2.66%	0.67%	Beta	
75–84	4.78%	1.20%	Beta	
85 and above	6.81%	1.70%	Beta	
Probability of developing an anxiety disorder	18.10%	0.70%	Beta	Kessler et al., 2005
**Relative Risk (RR)**				
RR of CVD for aircraft noise exposure	1.12	low: 1.07; high: 1.18	Triangular	Hansell et al., 2013
RR of anxiety for aircraft noise exposure	1.69	low: 1.00; high: 2.66	Triangular	Hardoy et al., 2005
RR of anxiety for CVD patients	1.66	low: 1.49; high: 1.82	Triangular	Fan et al., 2008
RR of CVD among those with prior CVD history	1.97	low: 1.67; high: 2.30	Triangular	Nichols et al., 2010

* For use in the Monte Carlo simulation.

**Table 2:** Costs (2016 U.S.$), incremental cost, quality-adjusted life years (QALYs) gained, incremental QALYs gained, and incremental cost-effectiveness (ICER) of changing the regulatory day–night average sound level (DNL) from 65 decibels (dB) to 55 dB versus the status quo.

Strategy	Cost	Incremental Cost	QALY	Incremental QALY	ICER *
Status quo	635,369		19.61		
Changing the regulatory DNL level from 65 dB to 55 dB	642,162	6793	20.22	0.61	11,163

* Incremental cost-effectiveness ratio was determined by cost (2016 U.S.$) per quality-adjusted life years (QALY) gained.

**Table 3:** One-way sensitivity analyses of the cost-effectiveness of changing the regulatory day–night average sound level (DNL) from 65 decibels (dB) to 55 dB versus the status quo.

Variable	Incremental Cost-Effectiveness Ratio *	
	Low	High
Relative risk (RR) of anxiety for aircraft noise exposure (Low: 1.00; High: 2.66)	195,717	Cost-saving
Cost of sound insulation (Low: −25%; High: +25%) ^†^	Cost-saving	24,448
RR of cardiovascular disease (CVD) for aircraft noise exposure (Low: 1.07; High: 1.18)	20,539	2822
Medical cost of anxiety (Low: −25%; High: +25%) ^†^	16,316	6010
Medical cost of CVD (Low: −25%; High: +25%) ^†^	15,931	6394
Probability of developing a CVD (Low: −25%; High: +25%)	13,830	7064
RR of anxiety for CVD patients (Low: 1.49; High: 1.82)	13,838	8912
Health utility decrement due to anxiety (Low: 0.117; High: 0.195)	13,914	9320
Productivity loss of CVD (Low: −25%; High: +25%) ^†^	12,860	9466

* Incremental cost-effectiveness ratio was determined by cost (2016 U.S.$) per quality-adjusted life years (QALY) gained; ^†^ We used ±25% for estimates with no random error, but that likely have geographic variation in costs. These high and low values roughly reflect the variation in the cost of living across localities in the United States.
